# Spatiotemporal Evolution of Carbon Emissions According to Major Function-Oriented Zones: A Case Study of Guangdong Province, China

**DOI:** 10.3390/ijerph20032075

**Published:** 2023-01-23

**Authors:** Jiang Zhu, Xiang Li, Huiming Huang, Xiangdong Yin, Jiangchun Yao, Tao Liu, Jiexuan Wu, Zhangcheng Chen

**Affiliations:** 1 Guangzhou Urban Planning and Design Survey Research Institute, Guangzhou 510060, China; 2 Guangdong Enterprise Key Laboratory for Urban Sensing, Monitoring, and Early Warning, Guangzhou 510060, China; 3 School of Architecture, South China University of Technology, Guangzhou 510641, China; 4 Marine Academy of Zhejiang Province, Hangzhou 310012, China

**Keywords:** carbon emissions, spatiotemporal characteristics, evolution analysis, major function-oriented zones, Guangdong Province

## Abstract

Studying the spatiotemporal evolution of carbon emissions from the perspective of major function-oriented zones (MFOZs) is crucial for making a carbon reduction policy. However, most previous research has ignored the spatial characteristics and MFOZ influence. Using statistical and spatial analysis tools, we explored the spatiotemporal characteristics of carbon emissions in Guangdong Province from 2001 to 2021. The following results were obtained: (1) Carbon emissions fluctuated from 2020 to 2021 because of COVID-19. (2) Over the last 20 years, the proportion of carbon emissions from urbanization development zones (UDZs) has gradually decreased, whereas those of the main agricultural production zones (MAPZs) and key ecological function zones (KEFZs) have increased. (3) Carbon emissions efficiency differed significantly among the three MFOZs. (4) Carbon emissions from coastal UDZs were increasingly apparent; however, the directional characteristics of MAPZ and KEFZ emissions were not remarkable. (5) Carbon transfer existed among the three kinds of MFOZs, resulting in the economy and carbon emissions being considerably misaligned across Guangdong Province. These results indicated that the MFOZ is noteworthy in revealing how carbon emissions evolved. Furthermore, spatiotemporal characteristics, especially spatial characteristics, can help formulate carbon reduction policies for realizing carbon peak and neutrality goals in Guangdong Province.

## 1. Introduction

It has been reported that greenhouse gas emissions (primarily CO_2_) are closely related to global climate change, and the increased frequency of extreme meteorological disasters [1], both of which severely affect sustainable development [2]. At the 75th United Nations Climate Conference, China announced that it would aim for carbon emissions peaking and neutrality by 2030 and 2060, respectively. To enable this, China must promote green, low-carbon, and high-quality developmental patterns, while mitigating the relationship between economic development and carbon emissions [3]. China is presently the global leader in carbon emissions; thus, it faces strong reduction pressure from the international community [4]. Specifically, identifying corresponding spatiotemporal characteristics is conducive to grasping the mechanisms of carbon emissions, and formulating reasonable carbon-reduction policies [5,6,7].

Guangdong Province serves as an important target region of carbon assessments for two reasons: (1) The specific characteristics and experiences of Guangdong Province can serve as a reference for the entire country with respect to policy generation. Presently, Guangdong is one of the most developed provinces in China, and exhibits considerable regional differences in development [8]; thus, it represents both a developed and developing area within China. For decades, the development patterns and experiences of Guangdong have acted as an important reference for national strategies compiled by the Central Government of China. In particular, Guangdong Province is in a key socioeconomic transformational stage. (2) Guangdong Province faces severe pressure to reduce carbon emissions while maintaining rates of economic development. According to the “Fourteenth Five-Year Plan” of the State Council of China, 2020-level carbon emissions per unit GDP in Guangdong should be decreased by 13.5% by 2025 [9,10]. Therefore, as the economy of Guangdong Province continues to grow, energy conservation and emissions reduction policies must be formulated to reduce carbon emissions levels. The main source of anthropogenically derived carbon emissions is fossil fuel energy combustion [11], which represented ~75% of the energy consumed in Guangdong Province in 2020 [12].

A General Spatial Land Planning plan for Guangdong Province (hereafter referred to as Guangdong Spatial Planning) is presently under preparation. This plan integrates the previous land use with urban planning, serving as the top-level policy for land development and ecological protection in the province [13]. Specifically, the major function-oriented zone (MFOZ) strategy is a spatial regulation measurement applied in Guangdong Spatial Planning to be used for specifying the future development orientation of each unit within the province. In Guangdong Spatial Planning, districts or counties serve as the smallest units, while the entire province is divided into three types of zones: urbanization development zones (UDZs), main agricultural production zones (MAPZs), and key ecological function zones (KEFZs). Basic information about each type of functional zone is shown in Table 1. In China’s planning system, most indicators (such as the quantity of cultivated lands) are subdivided at the county level for implementation and assessment [14]. Furthermore, most statistical data are collected at the county level; therefore, it is possible to implement carbon emissions reduction plans at this spatial scale across China. In a report on China’s contribution to emissions reductions submitted to the United Nations Framework Convention on Climate Change (UNFCCC), China proposed to employ “differentiated low-carbon development based on MFOZs” as an important policy tool for further reducing carbon emissions [15]. Therefore, studying carbon emissions from the MFOZ perspective enables the full use of county-level statistical data, as well as the ability to explore carbon emissions laws for different functional zones, and formulate reasonable emissions reduction policies.

Propositions aimed at carbon peaking and neutrality goals have a large impact on China’s environmental protection and industrial structure plans [16]. Based on these proposals, corresponding research in recent years has primarily focused on estimating carbon emissions, the possibility of achieving carbon peaking and carbon neutrality, in addition to the driving forces controlling carbon emissions. For example, Xu et al. [17] calculated the carbon emissions of Northeast China based on VIIRS nighttime lighting data, whereas Shi et al. [18] used DMSP-OLS night light data to estimate carbon emissions from the province and prefecture level within China from 1997 to 2012. Fang et al. [19] estimated the possibility of carbon peak by 2030 for 30 provinces in China. Guan et al. [20] and Zheng et al. [21] studied the driving forces of China’s carbon emissions during 2007–2016 and 1978–2018, respectively, and found that the contribution of fossil fuel energy consumption to carbon emissions had gradually decreased. Other studies have focused primarily on estimating or predicting the amount, rate of change, and factors influencing carbon emissions in China over the past and into the future [22,23]. To date, however, the spatiotemporal characteristics of carbon emissions remain poorly understood. Spatial emissions characteristics can reflect the distribution of the phenomenon and are, therefore, useful for understanding spatial heterogeneity [24,25]. Given the large differences in regional development in China, spatial characteristics must be considered when formulating important policies. Thus, studying the spatial characteristics of carbon emissions is crucial to improve the implementation of relevant policies [26].

Spatial characteristics of carbon emissions have been considered in studies that largely used night light data across a national or provincial scale [27]. Shi et al. [28] studied spatial changes in carbon emissions from counties along the Belt and Road based on DMSP/OLS night light data. Furthermore, Yang et al. [29] used DMSP/OLS night light remote sensing data to estimate carbon emissions and to analyze their distribution across Northeast China. Importantly, larger-scale studies are useful for understanding the general distribution of China’s carbon emissions from a macro-perspective; however, in the present situation where China’s carbon-reduction policies must be precisely implemented, more micro-analyses are essential for provincial-level policy formulation. Furthermore, night light data possess certain limitations, such as brightness saturation [30], multiple sets of satellite data from the same year [31,32], the incompatibility of image data from different satellite series [33], and a relatively high error of the fitting results (~20%) [17,18]. As fossil fuel combustion is the main source of anthropogenic carbon emissions, it would be more accurate to calculate emissions based on different amounts of energy consumed, with corresponding carbon emissions equivalence factors of each energy type [20].

China has recently proposed MFOZs as a new, but far-reaching spatial regulation method [13]; however, correlated studies have mainly focused on land use [34], regional development strategies [35], and national security [36]; whereas fewer studies have focused on carbon emissions. For example, Wang et al. estimated carbon emission transfer payments between different MFOZs in Guangdong [37].

In this study, statistical and geospatial analyses were used to evaluate the spatiotemporal characteristics of carbon emissions, and revealed how carbon emissions evolved from the perspective of MFOZs. It was found that the amount, efficiency, and spatial transfer of carbon emissions were constantly changing among the three kinds of MFOZs. This study’s novelty includes the following: (1) Previous studies have primarily focused on global- or national-scale emissions; however, the county-level emissions assessed here lay a foundation for finer-scale research. (2) Previous studies have mostly concentrated on the temporal characteristics of carbon emissions; however, spatial characteristics were also revealed here to better grasp for an enhanced perspective. (3) An improved understanding regarding how carbon emissions evolved from the perspective of MFOZs is provided here, representing an important spatial regulation metric in China.

The remainder of this paper is structured as follows: Section 2 presents an overview of the study area; Section 3 provides the data sources and research methods; Section 4 presents a detailed analysis of the spatiotemporal characteristics of carbon emissions in different MFOZs within Guangdong Province over 21 years (2001–2021); Section 5 discusses policy recommendations and future research directions; Section 6 presents the research conclusions.

## 2. Research Scope

**Figure 1:**
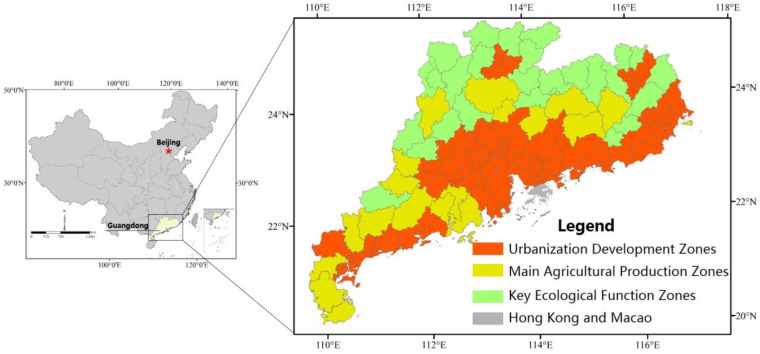
Scope of the study area.

Guangdong Province is located in southern China, and covers a total area of ~179,700 km^2^, comprising 122 county-level units (Figure 1). According to Guangdong Spatial Planning, the province has been divided into three MFOZ types: UDZs, MAPZs, and KEFZs. Of these, UDZs comprise 78 units, mainly located along the coastal areas. Comparatively, KEFZs comprise 25 units, mainly located in mountainous areas of northern Guangdong; whereas MAPZs comprise 19 interspersed units.

Over the decades, adjustments have been made to several county-level administrative divisions within Guangdong Province. This study used the 2019 administrative divisions of Guangdong Province as the standard to calculate energy consumption for each county-level unit; accordingly, errors may have been introduced regarding the amount of energy consumption for adjusted divisions, but the number of these units was small, and they were not expected to significantly impact the results.

## 3. Materials and Methods

### 3.1. Data Sources

Vector and statistical data were used in this investigation (Table 2), where the former included administrative division and location point data for high-energy-consumption enterprises. The MFOZ administrative region vector data were obtained from the Department of Natural Resources of Guangdong Province, as derived in 2019; therefore, the data time is 2019. Administrative division data were used for mapping and geospatial analyses. Specifically, points of cement, steel, power plants, and other high-energy-consumption enterprises in Guangdong Province were acquired from Gaode Online Map, which provided services after 2010. Accordingly, 2021 was chosen as the end-point for these data to correspond with the calculated carbon emissions results. The location points of the high-energy-consumption enterprises were used to generate kernel density maps and reflect corresponding changes across Guangdong Province from 2010 to 2021. County-level panel data were used for statistical analyses, including those related to various types of energy consumption and GDP. Specifically, these were derived from national Statistical Yearbooks, and used to calculate the carbon emissions of each county-level unit, as well as analyze the correlations between carbon emissions and GDP. Notably, China’s Statistical Yearbook in a given year contains data from the previous year; therefore, the Statistical Yearbooks from 2002 to 2022 pertain to the data from 2001 to 2021. As Statistical Yearbooks are published in October every year, the latest data available were from 2021.

### 3.2. Research Methods

#### 3.2.1. Carbon Emissions Calculations

The majority of anthropogenic CO_2_ emitted is derived from fossil fuel combustion [38]. Accordingly, carbon emissions in administrative regions can be more accurately calculated when the amount of fossil fuel use, and the carbon emissions coefficients (referring to the amount of CO_2_ emitted per kilogram of fuel consumed) for each type of fossil fuel are known [20,21]. To this end, the energy consumption of each county division was obtained from its respective Statistical Yearbook. Carbon emissions coefficients from various energy sources were based on guidelines for preparing provincial greenhouse gas inventories, as issued by the National Development and Reform Commission in 2011. This method for calculating carbon emissions was first proposed by the Intergovernmental Panel on Climate Change (IPCC) [20,21].

The method used to calculate carbon emissions is shown in Equation (1):

(1) $$CE=\overset{n}{\underset{i=1}{\sum }}Ti*Fi$$

where *CE* is the total carbon emissions of a unit in a given year, *n* is the number of energy types consumed, *Ti* is the amount of the *i*th energy type consumed by the unit in that year, and *Fi* is the CO_2_ emissions coefficient for the *i*th energy type. The carbon emissions coefficients for the energy types used are shown in Table 3, with all values confirmed by the National Development and Reform Commission of China. Notably, these values have been widely used tool for carbon emissions analyses in China.

#### 3.2.2. Methods for Analyzing the Evolution of Spatial Characteristics

**Figure 2:**
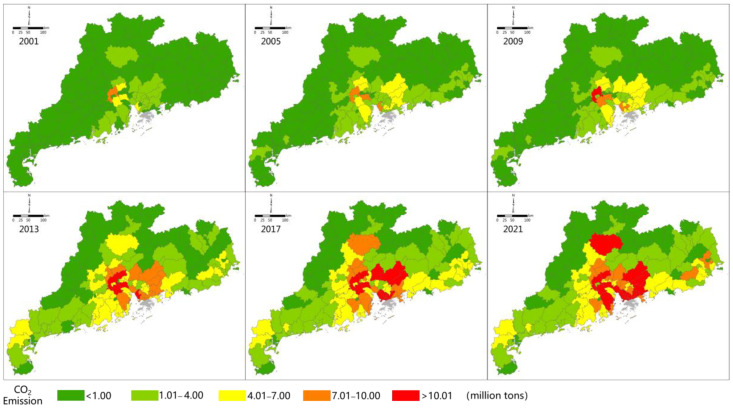
Time series of county-level carbon emissions from 2001 to 2021 across Guangdong Province.

Spatial characteristics are crucial for understanding how a phenomenon is distributed; thus, it has become increasingly valuable to inform government policy making. Unlike temporal characteristics, spatial characteristics are more difficult to express. In Figure 2, for example, spatial characteristics would generally be interpreted as follows: the outer section shows lower carbon emissions than the core, while the core portion shows more concentrated emissions characteristics. Such normal descriptions are far from sufficient for policy formulation, and more effective methods for grasping the precise spatial characteristics are required.

The gravity center and standard deviation ellipses are widely used to characterize spatial distributions of geographical phenomena [38]. In geography, the gravity center is conceptualized as the characteristic point of a geographical phenomenon’s spatial distribution, and the migration trajectory of the gravity center can be used to understand spatial distribution characteristics of geographical objects.

If the gravity center for carbon emissions shifts to the north, it indicates that the northern region contains a greater proportion of carbon emissions; whereas if the gravity center of carbon emissions and GDP are misaligned, it indicates that economic development does not match energy consumption. Moreover, if the standard deviation ellipses become “fatter”, it reflects the gradual dispersion of carbon emissions. These conclusions, which cannot be obtained through more simplified standardized descriptions, are critical for industrial and energy structural adjustments.

The gravity center of carbon emissions was calculated according to Equation (2) [40], and ArcGIS v.10.2 was used to calculate both the gravity center and GDP:

(2) $$\bar{x_{t}}=\frac{\sum_{i=1}^{n}I_{ti}X_{ti}}{\sum_{i=1}^{n}I_{ti}};\;\bar{y_{t}}=\frac{\sum_{i=1}^{n}I_{ti}Y_{ti}}{\sum_{i=1}^{n}I_{ti}}$$

where $\bar{x_{t}}$ and $\bar{y_{t}}$ are the abscissa and ordinate of the carbon emissions gravity center in year *t*, respectively; $I_{ti}$ is the carbon emissions of the *i*th unit in year *t*; $X_{ti}$ and $Y_{ti}$ are the abscissa and ordinate of the *i*th unit in year *t*, respectively.

The gravity center of a geographical element is also the center of its standard deviation ellipse [41]. Specifically, the standard deviation ellipse can quantitatively describe the concentration or dispersion of geographical elements in all directions through its parameters, such as the major and minor axes, as well as the angle of direction, while it reflects the overall outline, dominant distribution direction, and the spatial structure of these elements. The long semi-axis of the standard deviation ellipse represents the dominant distribution direction of geographical elements, while the short semi-axis represents the distribution range of data. The larger the ratio (oblateness) of the long and short semi-axes, the more notable the directivity of a geographical element, and the more concentrated the distribution. In contrast, when the long and short semi-axes are closer, the directivity of the data is less apparent. The equation for calculating the standard deviation ellipse is shown in Equation (3) [40], and ArcGIS *v.*10.2 was used to generate the standard deviation ellipses.

(3) $$SDE_{x}=\sqrt{\frac{\sum_{i=1}^{n}{\tilde{x}}_{i}{}^{2}}{n}},\;SDE_{y}=\sqrt{\frac{\sum_{i=1}^{n}{\tilde{y}}_{i}{}^{2}}{n}}$$

where ${\tilde{x}}_{i}=x_{i}-\bar{X}$, ${\tilde{y}}_{i}=y_{i}-\bar{Y}$, and *SDE_x_* and *SDE_y_* represent the lengths of standard deviation ellipses along the *x*- and *y*-axes, respectively.

Equation (4) was used to calculate the direction angle of the standard deviation ellipse:

(4) $$\tan\theta =\frac{A+B}{C}\;A=\sum_{i=1}^{n}{\tilde{x}}_{i}{}^{2}-\sum_{i=1}^{n}{\tilde{y}}_{i}{}^{2},\;B=\sqrt{A^{2}+4{\left(\sum_{i=1}^{n}{\tilde{x}}_{i}{\tilde{y}}_{i}\right)}^{2}},\;C=2\sum_{i=1}^{n}{\tilde{x}}_{i}{\tilde{y}}_{i}$$

where θ represents the direction angle of the standard deviation ellipse.

## 4. Results

In this study, county-level carbon emissions time series for Guangdong Province were generated from 2001 to 2021 (Figure 2), and these results were used to conduct carbon emissions research on sub-provincial-level scales. From the perspective of MFOZs, this study focused on the spatiotemporal evolutionary characteristics. Carbon Emission Accounts and Datasets (CEAD; www.ceads.net.cn) previously published provincial-scale emissions from 1997 to 2017 across China, and its data have been used in much research [42,43,44]. For validating the results, we compared the carbon emissions in 2017 from CEAD (514.64 million tons) and this study (559.08 million tons), and found the error to be ~8%, which is acceptable.

### 4.1. Temporal Analysis of Carbon Emissions of MFOZs

Temporal analyses were primarily conducted to study the evolution of carbon emissions over the study area. Total carbon emissions, growth rate, and efficiency were the three most fundamental temporal indicators used here. Accordingly, the total amount of carbon released reflected the general state of emissions, while growth rate trends represent the change over time, and the carbon efficiency reflects the relationship of carbon emissions and economy development. Through an analysis of these three indicators, the main temporal characteristics of carbon emissions were obtained for each MFOZ within Guangdong Province.

#### 4.1.1. Characteristics of Total Carbon Emissions and Growth Rate

**Figure 3:**
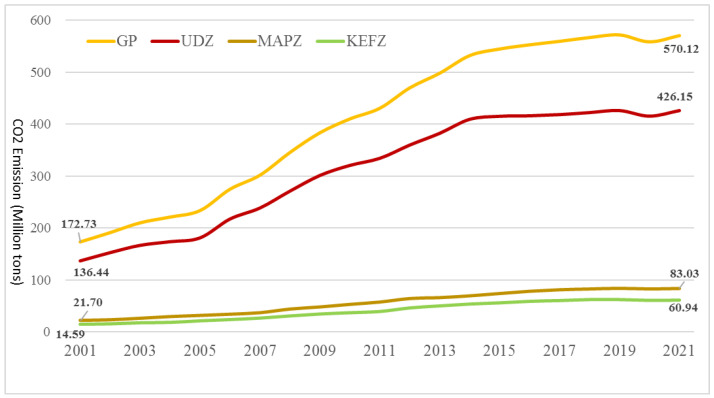
Changes in total carbon emissions from the three types of major function-oriented zones (MFOZs) in Guangdong Province from 2001 to 2021.

**Figure 4:**
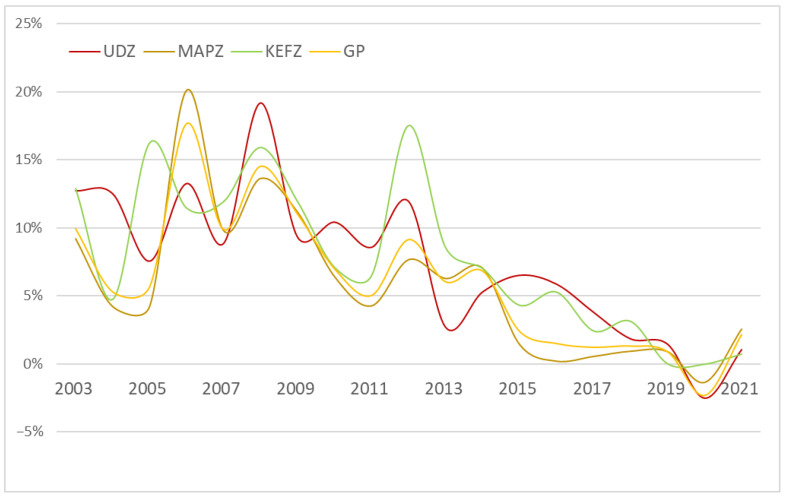
Changes in carbon emission growth rates from the three types of MFOZs in Guangdong Province from 2001 to 2021.

Figure 3 indicates the total provincial- and MFOZ-level amounts of carbon emitted in the study area. From 2001 to 2021, the total carbon emitted in Guangdong Province increased by 397.39 million tons (equivalent to 303.69%). Regarding the three MFOZs, emissions increased by 289.71 million tons for UDZs (287.08%), 61.33 million tons for MAPZs (365.77%), and 46.35 million tons for KEFZs (367.33%); however, the growth rate of carbon emissions for GD, UDZ, and MAPZ showed a fluctuating decline over time (Figure 4), including a peak in 2006 to 2008, after which they significantly decreased.

According to the “Analysis of Guangdong’s Energy Structure since the 12th Five Year Plan” [45], from 2010 to 2012, the GDP of energy-intensive industries accounted for 22.7% of the provincial total, 3.0% higher than that in 2010, with this change being most noticeable in northern Guangdong. Therefore, a temporary elevation of the carbon emissions growth rate was observed in 2012, with KEFZs showing the highest rates over the research period. The government of Guangdong Province rapidly noticed this phenomenon, and immediately issued a series of policies to restrict the development of energy-consuming enterprises. Therefore, the carbon emissions growth rate has not shown an apparent increase after 2012. Since the outbreak of COVID-19 in 2020, the three kinds of MFOZs showed apparent reductions in carbon emissions. Over the short term, non-essential economic activities have almost stopped, significantly reducing carbon emissions and improving environmental quality [46]. China has also enacted lockdown measures to combat the epidemic, drastically reducing industrial production and transportation. Further, the quarantined population at home has led to an additional significant reduction in carbon emissions over the short term. In 2021, the carbon emissions growth rate increased, as the majority of socioeconomic activities returned to normal as the pandemic was controlled. Notably, the resumption of work and implementation of economic stimulus plans during the post-pandemic period may lead to a rapid rebound in pollutants and carbon emissions [47]. Without a shift in economic growth patterns, any reductions in emissions from the recession will be temporary.

**Figure 5:**
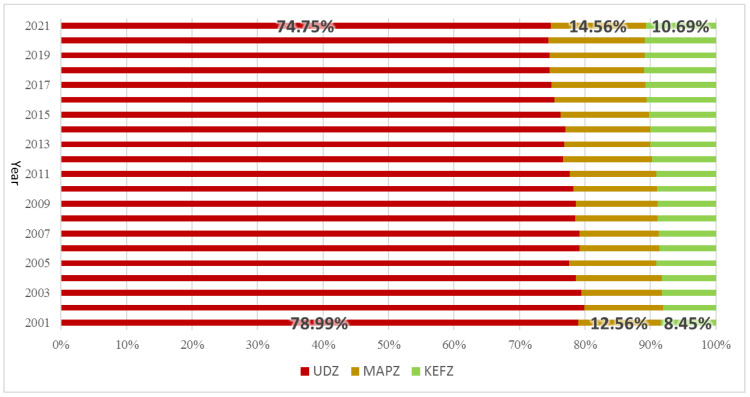
Changes in the proportion of carbon emitted from the three types of MFOZs in Guangdong Province from 2001 to 2021.

Rapid economic development and continuous population inflow rely heavily on fossil fuels [48]. In the study period, the proportion of carbon emitted from UDZs in the province was >74%, indicating that the correlated carbon emissions characteristics control the general provincial trend. The proportional amount emitted from UDZs decreased from 2001 (79.07%) to 2021 (74.75%), whereas the proportions emitted from MAPZs and KEFZs increased (Figure 5). This phenomenon implies that the average growth rate of carbon emissions in UDZs was lower than those in the other two functional zones over the 20-year period, as well as the fact that the implemented energy conservation and emissions reduction policies were more effective in UDZs than MAPZs or KEFZs. The impact of COVID-19 on the metropolis was far greater than those on small towns, potentially accelerating the reduction in carbon emissions proportions in UDZs.

#### 4.1.2. Carbon Emissions Efficiency Characteristics

**Figure 6:**
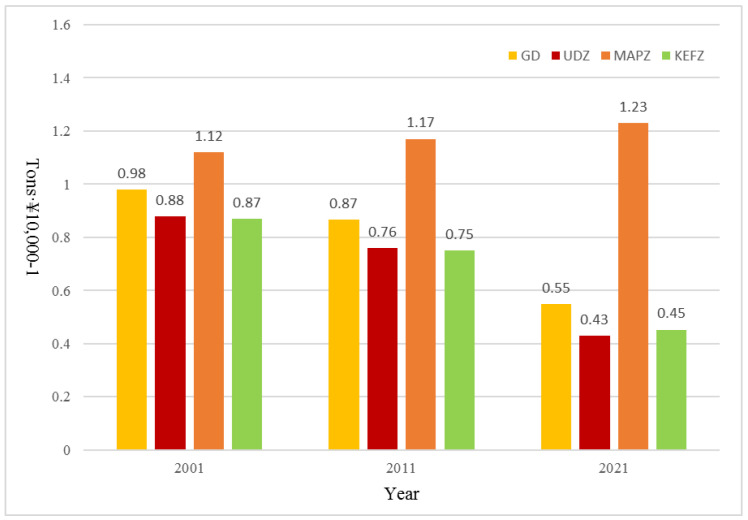
Changes in carbon emissions per unit GDP within Guangdong Province and for the three types of MFOZs from 2001 to 2021.

The carbon emissions efficiency of Guangdong Province improved over time (Figure 6), as emissions per unit GDP decreased from 0.98 tons·CNY 10,000^−1^ in 2001 to 0.55 tons·CNY 10,000^−1^ in 2021, representing a decrease of 43.87%. In addition, the decline from 2010 to 2021 was significantly greater than that from 2001 to 2010. This likely resulted from China joining the World Trade Organization (WTO) in 2001, allowing Guangdong Province to contribute to the global demand for industrial products. This was followed by the vigorous development of its chemical, cement, metal smelting, and other high-energy-consuming industries, thus providing large numbers of industrial products for China and other nations, thereby transforming China into a world factory over a relatively short period [49]. To support the power consumption of its factories, Guangdong Province built multiple thermal power plants between 2001 and 2005, which further increased carbon emissions [50]. Accordingly, the economic development of Guangdong Province from 2001 to 2008 largely came at the cost of resource consumption [51]; however, after 2010, China’s 12th Five-Year Plan proposed “resource conservation and green development” [52], as Guangdong Province explored a low-carbon development path, improving production technologies, eliminating backward production capacities, optimizing its industrial structure, and promoting clean energy use. Subsequently, from 2010 to 2021, the proportion of heavy industries in GDP within Guangdong Province decreased from 53% to 49%, and carbon emissions per unit GDP decreased significantly as well. Of the three types of MFOZs, the carbon emissions efficiencies of UDZs and KEFZs were higher, while those of MAPZs were lowest, reaching nearly half the average provincial level. This was likely because MAPZs not only provide a large amount of grain, vegetables, and fruits, but also acquired numerous industries transformed from the Pearl River Delta region. For example, Yingde County, which was classified as an MAPZ unit, produced ~30% of cement in Guangdong Province annually. It was also difficult for Guangdong’s MAPZs to realize the large-scale mechanized operations that occurred along China’s northern plain [53]; therefore, agricultural production in Guangdong’s MAPZs likely used comparatively more machine hours and associated amounts of fossil fuel, further reducing carbon emissions efficiency.

**Figure 7:**
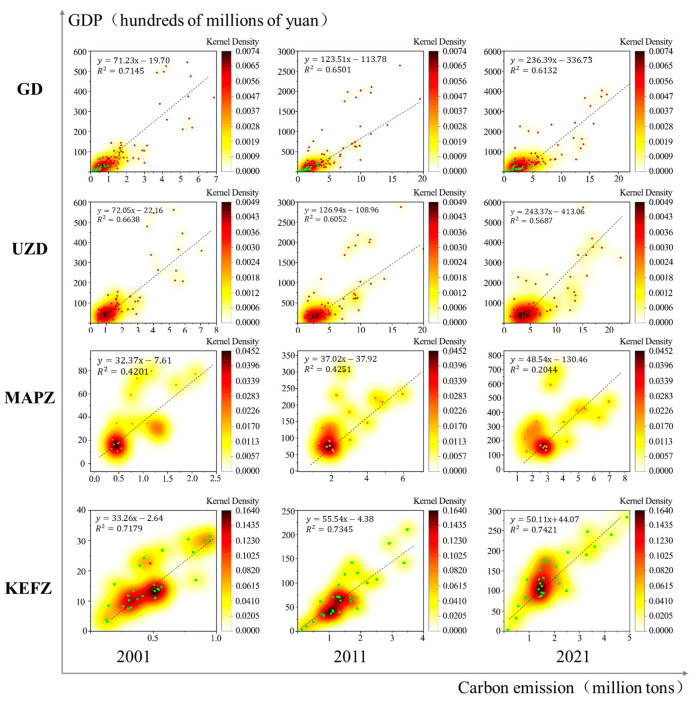
Correlation between carbon emissions and GDP in Guangdong Province and in the three types of MFOZs from 2001 to 2021.

To further study the carbon emission efficiency, a chart showing the correlation between carbon emissions and GDP was generated (Figure 7). On a provincial scale, the correlation coefficient between carbon emissions and GDP decreased over time, possibly due to increasing differences in the internal development patterns within Guangdong Province. The major cities of Guangdong have gradually achieved high-quality development, and their sustained economic development now depends less on fossil fuel energy; therefore, their corresponding development patterns are superior to those of other regions. The correlation coefficient between carbon emissions and GDP in the UDZs decreased from 0.66 in 2001 to 0.56 in 2021 due to the large developmental differences within these urbanized areas. The economic development of Guangzhou and Shenzhen was dominated by service industries, inherently relying less on fossil fuel energy resources than cities dominated by manufacturing and chemical industries (e.g., Dongguan and Foshan) [54]. The correlation coefficient between carbon emissions from MAPZs and GDP showed the most significant decline; thus, the carbon emissions efficiency of MAPZs requires urgent improvement. Lastly, the chart shows that the correlation coefficient between carbon emissions and GDP in KEFZs increased from 0.72 in 2001 to 0.74 in 2021, indicating that economic development in KEFZs was increasingly closely related to fossil fuel energy consumption. Notably, this phenomenon does not meet the high-quality development requirements of regions mainly engaged in primary industries and tourism.

### 4.2. Spatial Analysis of Carbon Emissions of MFOZs

#### 4.2.1. Changing Characteristics of the Standard Deviation Ellipse of Carbon Emission of MFOZs

The changes about oblateness of carbon emissions standard deviations, elliptical directional angle of carbon emissions standard deviations, and deviations in GDP and carbon emissions gravity center in Guangdong Province and its MFOZs are showed in Table 4, Table 5 and Table 6.

**Figure 8:**
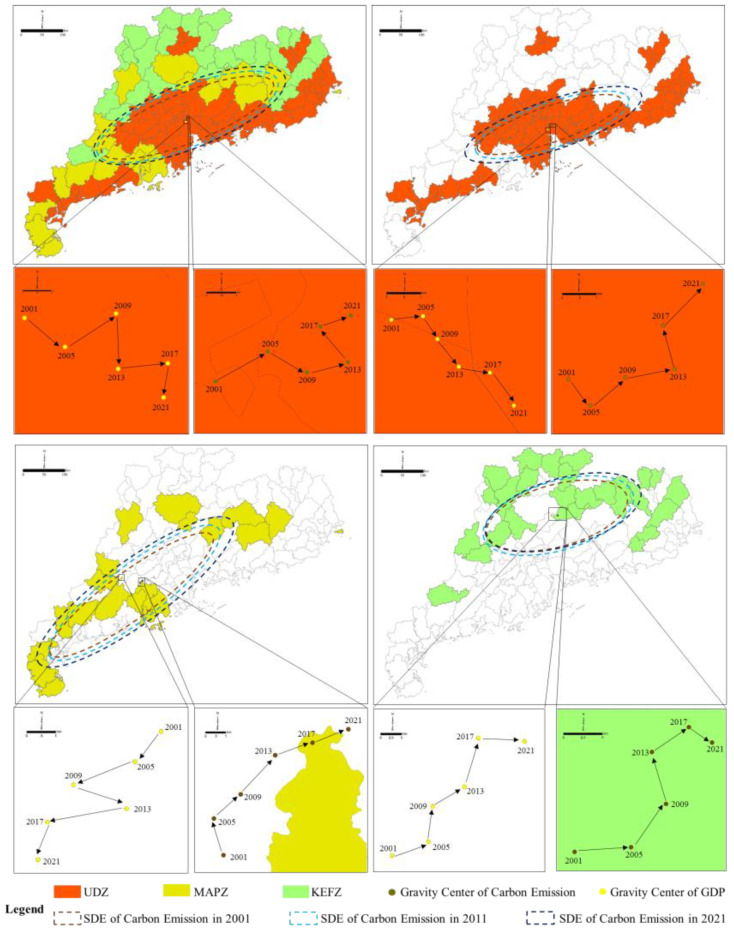
Migration of GDP, carbon emission (CE) gravity center, and changes in the standard deviation ellipse (SDE) in Guangdong Province and its MFOZs.

According to Figure 8, the primary axis of carbon emissions in Guangdong Province was roughly parallel to the coast. The oblateness of the standard deviation ellipse increased from 0.3709 in 2001 to 0.3773 in 2021, showing a gradual widening. This phenomenon indicates that carbon emissions in Guangdong Province became increasingly more distributed due to the increased proportion of carbon emissions from MAPZs and KEFZs perpendicular to the coastline. The directional angle of the standard deviation ellipse of carbon emissions in Guangdong Province decreased from 65.63° in 2001 to 64.72°, supporting that the spatial distribution of carbon emissions in Guangdong Province moved roughly to the north.

The axis of the standard deviation ellipse of carbon emissions in UDZs was roughly the same as that of Guangdong Province. The oblateness of the standard deviation ellipse decreased from 0.3291 in 2001 to 0.3233 in 2021, indicating that carbon emissions in the urbanization development zone continued to gather along the coastline; whereas the direction angle increased from 66.79° in 2001 to 67.47° in 2021.

The MAPZ units were scattered throughout Guangdong Province, and their carbon emissions standard deviations spanned a large range. From 2001 to 2021, the oblateness of the standard deviation ellipses of carbon emissions in MAPZs increased from 0.3152 to 0.3074, implying that the distribution of corresponding emissions became increasingly dispersed, with a consistent reduction in their obvious directivity. Further, the direction angle increased slightly from 52.65° in 2001 to 53.43° in 2021.

The carbon emission axis of the KEFZs essentially followed that of the northern mountains in Guangdong. From 2001 to 2021, the oblateness of the standard deviation of carbon emissions increased from 0.4126 to 0.4018, indicating that the directivity of carbon emissions transitioned from less to more obvious. This may have been related to KEFZs having similar developmental patterns, where the developmental stage of each unit was relatively small. Therefore, no obvious growth direction of carbon emissions was observed. Additionally, the orientation angle of the standard deviation ellipse increased slightly from 78.73° in 2001 to 79.25° in 2021.

#### 4.2.2. Gravity Center Migration of Carbon Emissions and GDP of MFOZs

Economic development is one of the main forces driving carbon emissions; thus, future carbon emissions reduction policies are intrinsically related to economic advancements and industrial adjustments [55]. Therefore, the migration of the carbon emissions center and the economic (GDP) center for each county-level unit were calculated to determine correlations between the carbon emissions from different MFOZs.

From 2001 to 2021, the GDP gravity center in Guangdong Province moved consistently toward Shenzhen, with an average annual movement of ~0.8 km·yr^−1^. This indicated that the economic growth rate of the Shenzhen metropolitan area was higher than other regions. The carbon emissions gravity center of Guangdong Province first shifted to the southeast, and then to the northwest, with an average annual movement of ~1.2 km. This occurred because prior to 2008, the pillar industries in Guangzhou and Shenzhen (with rapidly developing economies) were more dependent upon resource consumption associated with higher carbon emissions [56]. Accordingly, the carbon emissions gravity center moved consistently to the southeast; however, after 2008, cities such as Guangzhou and Shenzhen upgraded their industrial structures, optimized their low-end production capacity, and implemented a green development pattern. Thus, the proportion of carbon emissions decreased compared to those in other regions. The resulting effect was that the gravity center of carbon emissions in Guangdong Province moved north after 2008, away from its core areas.

**Figure 9:**
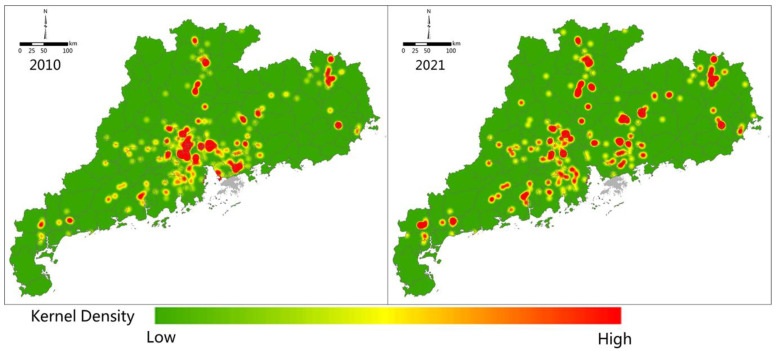
Kernel density of high-energy-consuming enterprises (such as cement, metallurgy, petrochemical industries, and thermal power plants) over Guangdong Province in 2010 and 2021.

**Figure 10:**
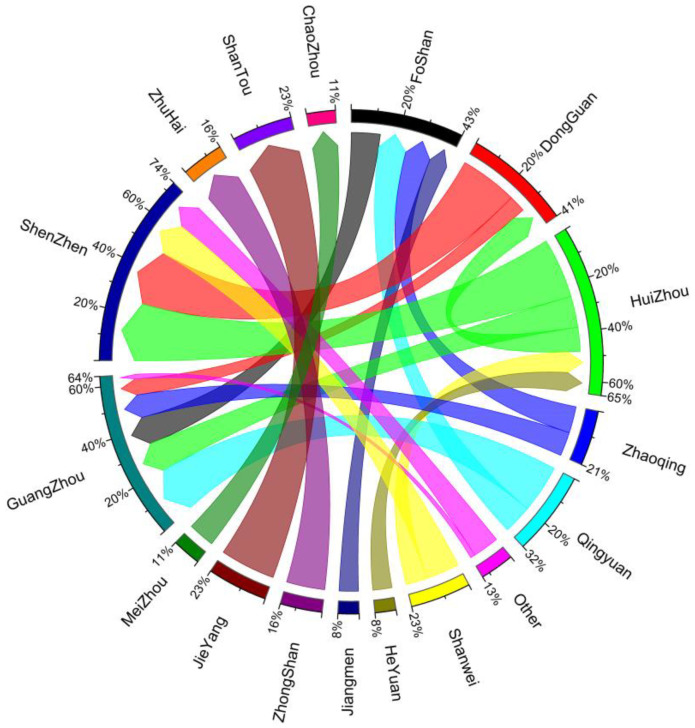
Power transfer of Guangdong Province in 2021.

A further analysis showed that the deviation in economic and carbon emissions gravity centers in Guangdong Province increased continuously from 9.67 km in 2001 to 11.66 km in 2021. During the industrial upgrading process, the main cities of Guangdong Province gradually moved their energy-consuming industries (e.g., power plants, metallurgy, petrochemical, and cement industries) to northern and western provincial regions [57]. The spatial kernel density of high-energy-consuming enterprises were also calculated based on location-point data from 2010 to 2021 (Figure 9). The results showed that from 2010 to 2021, the number of energy-consuming enterprises in the Pearl River Delta region decreased, whereas those in MAPZs or KEFZs (such as in Yingde County) increased significantly. Although industry transfer supported local economic development, there was a marked increase in local carbon emissions observed. As the carbon emissions trading mechanism had not been fully established, developed regions in Guangdong did not pay a carbon tax, nor any other ecological compensation to the place of production when purchasing and using products that generated significant amounts of CO_2_ during production. Using the power consumption in Guangzhou and Shenzhen as an example, the corresponding self-sufficiency rates of power decreased over time, reaching 36.22% and 24.58%, respectively, in 2021. Further, Qingyuan and Huizhou had become the largest power sources in Guangzhou and Shenzhen by 2021 (Figure 10); therefore, a carbon transfer phenomenon occurred during the process of economic development within the province, and the carbon source that originally existed in the core cities flowed from the UDZs to the MAPZs and KEFZs. This transfer of industries promoted both economic development and increased local carbon emissions.

The migration characteristics of the GDP and carbon emissions gravity centers in UDZs were similar to those of Guangdong Province. The GDP centers continued to move to the southeast (toward Shenzhen), with an average annual movement rate of ~1 km·yr^−1^, illustrating the existence of differences in economic development within the UDZs. Here, the carbon emissions gravity center underwent a process of moving first to the southeast, and then to the north, whereas the deviation of the economic and carbon emissions gravity centers gradually increased from 11.58 km in 2001 to 15.78 km in 2021.

Comparatively, the GDP and carbon emissions gravity centers for MAPZs moved in opposite directions. The economic gravity center continued to move to the southwest at ~1.1 km·yr^−1^. This occurred because of the natural conditions in western Guangdong, as the agricultural development levels here are superior to those in the north. The carbon emissions gravity center moved toward the northwest at an average annual rate of ~1.4 km·yr^−1^, likely because the MAPZs in northern Guangdong undertook more energy-consuming industries in the Pearl River Delta region, with corresponding greater carbon emissions. The deviation in the economic and carbon emissions gravity centers in MAPZs was the largest among the MFOZs, at ~50 km.

The GDP and carbon emissions gravity centers of KEFZs moved roughly toward the northeast, whereas the average annual movements of the economic and carbon emissions centers of gravity were approximately 1.5 and 1.3 km·yr^−1^, respectively, with an ~15 km deviation between the two.

## 5. Discussion

According to results, COVID-19 led to a temporary reduction in carbon emissions during 2020 [58]; however, influenced by the international pandemic, the development of more clean energy projects, such as wind and solar, was put on hold across the globe, leading to a deeper global reliance on fossil fuel energy sources, including natural gas and coal. The path of carbon peaking and neutralization in the post-pandemic era may be more complicated, as this situation may place more severe carbon reduction pressures on Guangdong Province. The present study indicated that this province will maintain high total carbon emissions into the future, while the spatial deviance of economic development and carbon emissions may become more apparent. Industrial production and urban life are the major sources of carbon emissions, while improving carbon emissions efficiency is key to balancing economic development and fossil fuel consumption [59,60]. In the context of a post-COVID-19 era, as well as carbon peaking and neutralization across China, Guangdong Province is under severe pressure; accordingly, the following policy recommendations have been proposed:

1. Continuous upgrading of the energy infrastructure is necessary for Guangdong Province to reduce fossil fuel consumption. Thermal power plants consume a large amount of coal in China, contributing to ~50% of national carbon emissions each year; however, Guangdong Province is home to various types of clean energy resources. UDZs need to fully implement nuclear energy, as coastal areas should encourage wind energy development, and KEFZs can capitalize on hydro resources. This greater use of clean energy to generate electricity will reduce coal consumption, thus decreasing carbon emissions; however, due to an imperfect grid infrastructure, the cost of these green resources is significantly higher than more traditional thermal power in some regions, severely limiting the promotion of green electricity. Accordingly, Guangdong should continue to upgrade the energy infrastructure to reduce green power costs [56].
2. Optimizing the industrial transfer and development patterns is crucial to the reduction in carbon sources in MAPZs and KEFZs. The migration of industries from core areas of Guangdong Province has assisted other regions; however, this assistance also resulted in a strong carbon transfer effect, where although local GDP has increased, local carbon emissions and environmental damages have as well. Carbon transfer is not unacceptable across a provincial scale, and transferring carbon emissions from UDZs to MAPZs or KEFZ, which maintain more dense vegetation cover, is more conducive to carbon fixation [61]. Therefore, when core area industries are transferred, they should simultaneously export advanced production processes and energy-saving technologies to these developing regions to ensure the promotion of more sustainable, high-quality development [62].
3. It remains necessary to immediately formulate carbon emissions reduction plans, or establish regional ecological compensation systems. According to Wang et al. [63], Guangdong will not have reached maximum carbon emissions by 2030; therefore, the provincial government must immediately formulate a carbon emissions reduction plan by defining the necessary tasks for each county-level unit. For regions that have not yet completed industrial upgrading, and still are more reliant upon higher-energy consumption and carbon emissions industries, a forced reduction in emissions can deprive them of their economic developmental rights. Therefore, when formulating carbon emissions reduction strategies, one must refer to current carbon emissions, investigate the precise carbon emissions source, and consider carbon transfer effects for any comprehensive emissions reduction plans. Furthermore, Guangdong Province should establish an ecological compensation system for carbon, and increase the price of high-carbon-emissions industrial products (e.g., cement, thermal power, and steel) in consideration of carbon fixation costs [64]. Lastly, according to the principle of carbon neutralization within administrative areas, UDZs should pay corresponding carbon emissions compensation fees annually to MAPZs or KEFZs.

This study’s main limitation was that some indirect carbon emissions were ignored. Here, carbon emissions were calculated according to fossil fuel use and carbon emissions coefficients. While this method can accurately account for the majority of the carbon emissions, various indirect emissions sources are excluded [65], such as those caused by land-use [66], industrial production [67], and urban operation [68].

Future analyses should be directed at the following improvements. (1) More data can be integrated to develop a better carbon emissions approach. (2) Carbon emissions after 2021 need be continuously evaluated to study their continued evolution in a post-COVID-19 era. (3) The possibility for carbon peaking and neutrality of different MFOZs can be more precisely assessed.

## 6. Conclusions

This study calculated the carbon emissions of 122 counties or districts in Guangdong Province from 2001 to 2021, and analyzed the corresponding spatiotemporal characteristics from the perspective of MFOZs. The main conclusions are as follows:

(1) Between 2001 and 2021, the total carbon emissions from Guangdong Province increased by 397.39 million tons (equivalent to 303.69%). Further, COVID-19 showed a marked influence on carbon emissions during 2020 and 2021. Moreover, a strong relationship between MFOZs and carbon emissions was observed. UDZs were responsible for >74% of the annual carbon emissions in Guangdong Province; however, over the 20-year analysis period, the proportion of carbon emitted from UDZs slightly decreased, while those of MAPZs and KEFZs increased. The change in carbon emissions proportions among MFOZs can potentially provide an important reference for the development of more directed carbon reductions policies.
(2) Between 2001 and 2021, the carbon emitted per unit GDP of Guangdong Province decreased from 0.98 tons·CNY 10,000^−1^ to 0.55 tons·CNY 10,000^−1^, with the annual patterns indicating a continuous increase in the carbon emissions efficiency. Differences were also evident between the carbon emissions efficiencies of MFOZs units, where those of UDZs and KEFZs improved, being markedly higher than those of MAPZs. The acquisition of numerous low-end industries may be the main reason why the carbon emissions efficiencies in MAPZs were substantially lower.
(3) From 2001 to 2021, the range of the standard deviation ellipse for carbon emissions in Guangdong Province, and its corresponding MFOZs gradually increased. Specifically, carbon emissions from UDZs were more strongly distributed along the coastline, while those of MAPZs and KEFZs became increasingly less dominant.
(4) Transforming traditional industries created a carbon transfer pattern across Guangdong Province. From 2001 to 2021, the GDP and carbon emissions gravity centers and its MFOZs deviated to varying degrees. The GDP gravity center of UDZs continually moved toward Shenzhen, while the carbon emissions gravity center moved from south to north. The GDP and carbon emissions gravity centers of MAPZs gradually moved in opposite directions, and the deviance between them was the largest of the three MFOZ types. Lastly, the migration directions of GDP and carbon emissions gravity centers for the KEFZs showed similar patterns. The deviance between the economic and carbon emissions gravity centers may be a key issue to address in future carbon emissions reduction plans of Guangdong Province, and should be explored further.

## Figures and Tables

**Table 1:** Basic information on the major function-oriented zones (MFOZs) in Guangdong Province [13].

Category	Number	Concept
Urbanization development zones (UDZs)	78	Zones have good economic and social development foundations, a large population, and good industrial capacity. These areas maintain the power to promote high-quality development, are leaders in regional socioeconomic development, and provide important support for promoting regional coordinated development.
Main agricultural production zones (MAPZs)	19	Zones comprise large areas of agricultural land, or possess good agricultural development conditions. They can guarantee national food supplies, and are providers of important agricultural products. This zone type functions as a key national construction zone for agricultural production, ensuring the safety of agricultural products, with strong potential for modern agricultural construction, as well as showing effective agricultural product processing and ecologically sound industries, in addition to providing a specific economy for the county.
Key ecological function zones (KEFZs)	25	Zones provide important ecological services, but have a fragile ecological structure. Their functional orientation is to ensure national ecological security, maintain ecosystem services, promote the governance of various natural resource systems, as well as maintain and improve the supply capacity of ecological products.

**Table 2:** Data sources used in this study.

Category	Name	Source	Time
*Vector*	Major function-oriented zone (MFOZ) administrative regions in Guangdong Province;	Department of Natural Resources of Guangdong Province	2019
	Points of cement, steel, power plants, and other high-energy-consumption enterprises in Guangdong Province;	Gaode Online Map	2010, 2021
*Statistical*	Energy consumption data for each county-level unit in Guangdong Province;	Statistical Yearbook of County-level units in Guangdong Province	2002–2022
	GDP data of county-level units in Guangdong Province		

**Table 3:** CO_2_ emission coefficients of various energy sources [39].

Energy Type	Coal	Coke	Crude	Gasoline	Kerosene	Diesel	Fuel	Natural Gas
CO_2_ emissions coefficient (kgCO_2_·kg^−1^)	1.9003	2.8604	3.0202	2.9251	3.0719	3.0959	3.1705	2.1622

**Table 4:** Changes in the oblateness of carbon emissions standard deviations in Guangdong Province and its MFOZs from 2001 to 2021.

Year	GD	UDZ	MAPZ	KEFZ
2001	0.3709	0.3291	0.3152	0.4126
2005	0.3720	0.3277	0.3131	0.4086
2009	0.3726	0.3265	0.3128	0.4085
2013	0.3741	0.3256	0.3093	0.4075
2017	0.3758	0.3242	0.3081	0.4045
2021	0.3773	0.3233	0.3074	0.4018

GD, Guangdong Province; UDZ, urbanization development zone; MAPZ, main agricultural production zone; KEFZ, key ecological function zone.

**Table 5:** Changes in the elliptical directional angle of carbon emissions standard deviations in Guangdong Province and its MFOZs from 2001 to 2021.

Year	GD	UDZ	MAPZ	KEFZ
2001	65.63°	66.79°	52.65°	78.73°
2005	65.15°	66.91°	52.76°	78.86°
2009	65.03°	66.97°	53.10°	79.07°
2013	64.88°	67.20°	53.21°	79.12°
2017	64.84°	67.33°	53.40°	79.23°
2021	64.72°	67.47°	53.43°	79.25°

**Table 6:** Deviations in GDP and carbon emissions gravity center (km) across Guangdong Province and its MFOZs from 2001 to 2021.

Year	GD	UDZ	MAPZ	KEFZ
2001	9.67	11.58	47.40	14.53
2005	10.86	10.69	47.26	14.54
2009	10.33	11.22	45.71	15.21
2013	10.93	13.45	47.18	14.20
2017	11.38	13.37	48.37	14.23
2021	11.66	15.78	49.79	14.66

## Data Availability

The data presented in this study are available upon request to the corresponding author.
